# Large-scale Direct Targeting for Drug Repositioning and Discovery

**DOI:** 10.1038/srep11970

**Published:** 2015-07-09

**Authors:** Chunli Zheng, Zihu Guo, Chao Huang, Ziyin Wu, Yan Li, Xuetong Chen, Yingxue Fu, Jinlong Ru, Piar Ali Shar, Yuan Wang, Yonghua Wang

**Affiliations:** 1 Bioinformatics Center, College of Life Sciences, Northwest A&F University, Yangling, Shaanxi, 712100, China; 2 Department of Materials Science and Chemical Engineering, Dalian University of Technology, Dalian, Liaoning, 116000, China; 3 Department of Pathology and MCW Cancer Center, Medical College of Wisconsin, Milwaukee, WI 53226. USA

## Abstract

A system-level identification of drug-target direct interactions is vital to drug repositioning and discovery. However, the biological means on a large scale remains challenging and expensive even nowadays. The available computational models mainly focus on predicting indirect interactions or direct interactions on a small scale. To address these problems, in this work, a novel algorithm termed weighted ensemble similarity (WES) has been developed to identify drug direct targets based on a large-scale of 98,327 drug-target relationships. WES includes: (1) identifying the key ligand structural features that are highly-related to the pharmacological properties in a framework of ensemble; (2) determining a drug’s affiliation of a target by evaluation of the overall similarity (ensemble) rather than a single ligand judgment; and (3) integrating the standardized ensemble similarities (*Z score*) by Bayesian network and multi-variate kernel approach to make predictions. All these lead WES to predict drug direct targets with external and experimental test accuracies of 70% and 71%, respectively. This shows that the WES method provides a potential *in silico* model for drug repositioning and discovery.

A system-level understanding of the relationships between drugs and their targets, especially direct targets^1^, is vital to address the efficacy and safety-related issues of compounds in the later stages of drug discovery and development^2^,^3^ and, thus, to reduce the high attrition rates in clinical trials^4^. Various biological means are available for identifying drug targets^5^,^6^,^7^, but the detection on a large scale remains challenging and expensive even nowadays. The obstacle towards this goal lies in the time and costs of pharmacological experiments that can accurately recapitulate the target response for diverse drugs^8^.

Recently, many experiment-based approaches including the high-density microarray and cell-based assays have been proposed to investigate the indirect or direct features of drug–target interactions^8^,^9^. However, the most reliable evidence of the direct interactions is the co-crystallization of the target proteins with drugs in a solution^10^. Recent developments in biotechnology have contributed to the increase in the amounts of high-throughput data for drugs and targets in the omics level, which can be precious sources for recognizing unknown drug-target interactions^11^. These also accelerate a variety of *in silico* approaches that have been developed for predicting potential targets. A simple way to measure direct the interactions might be the molecular docking simulation^12^, but which is limited by the availability of a reliable three dimensional (3D) structure of target proteins^13^. Thus, it is still very important to develop efficient computational methods to predict drug targets, which are independent of the protein structures.

Our previous work has developed a chemogenomic model based on chemical, genomic, and pharmacological information for characterizing the complicated interactions between ligands and targets^14^. However, due to the limitation of database used, this model could not discriminate those direct or indirect interactions. Another recently developed similarity ensemble approach (SEA) is capable of detecting the direct interactions based on the chemical similarity of ligand sets, which has been demonstrated as an effective conceptual and methodological breakthrough in this field^15^.

In this work, we propose a novel weighted ensemble similarity (WES) algorithm, an extension of the SEA method, to predict the drug-target direct interactions. Here, the term ensemble is an extension concept derived from statistical physics. As we know, each protein (receptor) has several ligands, these ligands construct a set, and here, the set was treated as an ensemble. This concept is proposed based on the following considerations: (1) if the ligand set has structurally similar compounds, then the ensemble average will cover a narrow chemical space. Thus, to compare a compound with the ensemble average or any single compound in a set might be have similar results; (2) however, in most cases, the ligands are diverse for a receptor like P-glycoprotein^16^ or COX2^3^, they might be divided into several smaller sub-clusters. If the prediction of a compound that is still made based on its similarity with a certain compound in the training set, it will not give reliable results. Thus, a more reasonable way is to compare a compound similarity with the whole feature of an ensemble (set).

Here, the WES model was built on a large data set involving 98,327 drug-target relations, which includes BindingDB^17^ (http://www.bindingdb.org/bind/index.jsp, access time: January 16, 2014), Drugbank^18^ (http://www.drugbank.ca/, access time: January 16, 2014), PDB^19^ (http://www.rcsb.org/pdb/, access time: January 16, 2014) databases, and GoPubMed (http://www.ncbi.nlm.nih.gov/, access time: January 30, 2014). The efficiency of the model was also compared with other published models and further validated by pharmacological experiments.

## Results

### WES—an algorithm for predicting direct interactions of drugs and targets

The algorithm works in three phases: (1) identifying the key ligand structural and physicochemical features (CDK and Dragon) that are highly-related to the pharmacological properties in a framework of ensemble. We assembled the feature matrix for the ligand set of each protein based on statistical tests (non-parametric Wilcoxon Sum Rank Test for Dragon feature; one-sided Fisher’s exact test for CDK feature). (2) Determining a drug’s affiliation of a target by evaluation of the overall similarity of an ensemble rather than a single ligand judgment. As the resulting score does not discriminate relevant similarities from random but depends on the number of ligands in each set, it is not a perfect assessment of the overall similarity of the ligand sets. Then the overall similarities were converted into the size-bias-free normalized values to eliminate the relevant similarities from random. (3) And finally, integrating the standardized ensemble similarities (*Z score*) by Bayesian network to make predictions.

## Model performance.

###  

**Figure 1:**
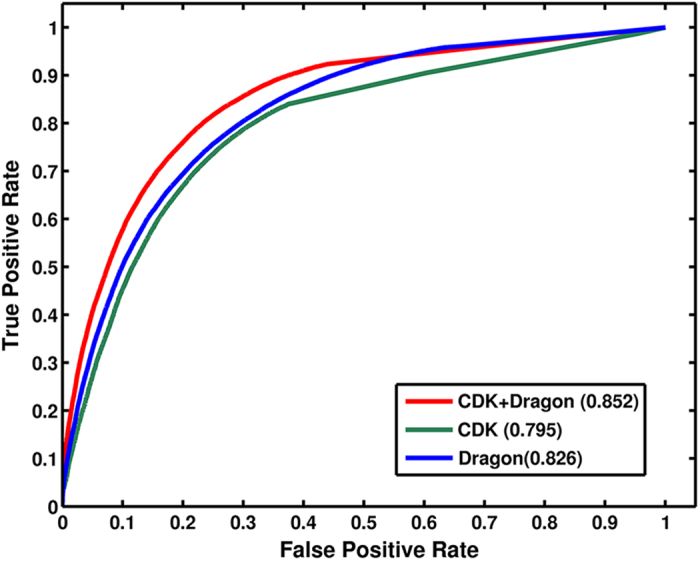
The performance of the WES model based on CDK, Dragon, and CDK-Dragon features.

Feature analysis. To investigate the effects of different structural features of the ligands on the model performance, we have used the Chemical Development Kit (CDK), Dragon and the CDK-Dragon hybrid features for model construction, respectively (see Methods for details). Table 1 illustrates the results in terms of precision and recall rates. Clearly, the hybrid model outperforms both the CDK and Dragon ones in recovering the negative links. Notably, the hybrid model for the leave-one-out cross-validation (LOOCV) performs well in predicting the binding (sensitivity 85%, SEN) and the non-binding (specificity 71%, SPE) patterns, with the accuracy of 78%, the precision (PRE 74%) and the area under the receiver operating curves (AUC) of 0.85, respectively. It is noted that all the scores (*Z score* for CDK and Dragon model and likelihood for CDK-Dragon hybrid model), used to make prediction, in this work were selected when the models achieve the highest *F1 score* in cross-validation otherwise specified (see Methods for details). The ROC curves (Fig. 1) show that all the three models are capable of catching sufficient information related to detect interactions at high true-positive rates against low false-positive rates at any threshold. With the increase of the AUC in the complete dataset, the hybrid model improves the ability to identify those known drug-target links, demonstrating that more chemical and pharmacological information introduced to build models can achieve better predictive activity.

To investigate the influence of weighted features attributed to the WES performance, we tested the different inputs: weighted features vs. non-weighted features. Table S1 shows that the weighted hybrid feature-based WES outperforms the non-weighted feature-based model, with the ACC of 78%, PRE of 74% and AUC of 0.85, respectively. This reflects that WES algorithm weights and selects features to reduce dimensionality of the descriptor set, thus resulting in good performance.

Also we have made a check of the effectiveness of integrating the standardized ensemble similarities (*Z score*) by Bayesian network. Notably, the integrated WES model also performs better than the non-integrated one in predicting the binding (SEN 85%) and the non-binding (SPE 71%) patterns (Table S1). These results serve to highlight the fact that integration procedure of WES algorithm exhibits high prediction efficiency.

### External data validation

To ensure the reliability of the WES model, we further carried out an external validation. The dataset for external validation includes both the binding (positive sample) and non-binding data (negative sample) as following: 1) the positive samples were extracted from PDB for those ligand-protein pairs with the half-maximal inhibitory concentrations (IC_50_) < 10 μM. The interactions which overlap with the training set for model construction were manually deleted, and finally 649 interactions were obtained; 2) the negative samples were achieved from BindingDB with a filter criterion of IC_50_ > 500 μM. And finally, 3,172 ligand-target non-binding data was obtained as negative samples. The hybrid model shows the prediction ACC of 71% (458/649) for the positive samples and 70% (2,209/3,172) for the negative samples. All these demonstrate the weighted hybrid WES achieves excellent performance for different data sources.

### Target class prediction

The performance of WES method was further tested on five pharmaceutical classes involving enzymes (n = 761), ion channels (n = 78), membrane proteins (n = 275), transporters (n = 50) and transcription factors (n = 39), respectively. Figure 1 and Table 1 show the AUC, SEN, SPE, PRE and ACC of the models. WES displays the highest prediction ability for the transcription factor (ACC = 0.80) and the membrane protein (ACC = 0.79), followed by the enzyme (ACC = 0.78), transporter (ACC = 0.79) and ion channels (ACC = 0.75), respectively.

Also, we have compared the performance of WES optimal model for target class prediction with other published models (enzymes, 664; ion channels, 204; membrane proteins, 95; nuclear receptors, 26; respectively.), including the nearest profile, weighted profile, bipartite Graph learning methods and the same criteria^5^. Table 2 indicates that all the methods have quite high AUC and SPE but low SEN values. The WES and bipartite graph model outperform the other two models (nearest profile, weighted profile). However, it has to be noted that, the WES model was constructed with a lager dataset exhibiting more molecular and pharmacological diversities, thus it is believed that WES might have more generalization ability for making predictions.

### Comparison of WES with 1NN

**Figure 2:**
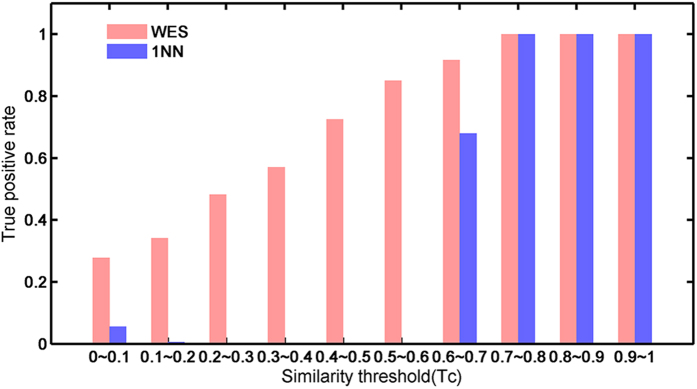
Comparsion of WES with 1NN. The ture positive rate of WES (red) and 1NN (blue) are shown as bars along with the similarity bins (x-axis).

In multi-objective pattern recognition, the k-Nearest Neighbors algorithm (k-NN) is a non-parametric and widely used method. The output depends on whether k-NN is used for classification by a majority vote of its neighbors, with the object being assigned to the class most common among its k nearest neighbors (k is a positive integer, typically small). WES has been compared to a one nearest neighbor (1NN) model (Fig. 2), which judges the probability of a drug targeting to a protein based only on the maximum similarity to the reference ligands of the target. For close analogs, Tanimoto coefficients (Tc) > 0.65, the fraction of true positives was comparable between 1NN and WES (Fig. 2). Surprisingly, by across most similarity thresholds, WES substantially outperforms 1NN. Notably, among the correct drug-target predictions by WES, 4,319 of them show low similarity (Tc < 0.4) with the ligand sets of their respective targets. However, the proportion held by 1NN is zero. These results prove that WES is more capable of predicting drug targets for various structurally diverse chemicals.

### Evaluation of ligand scaffold hopping

**Figure 3:**
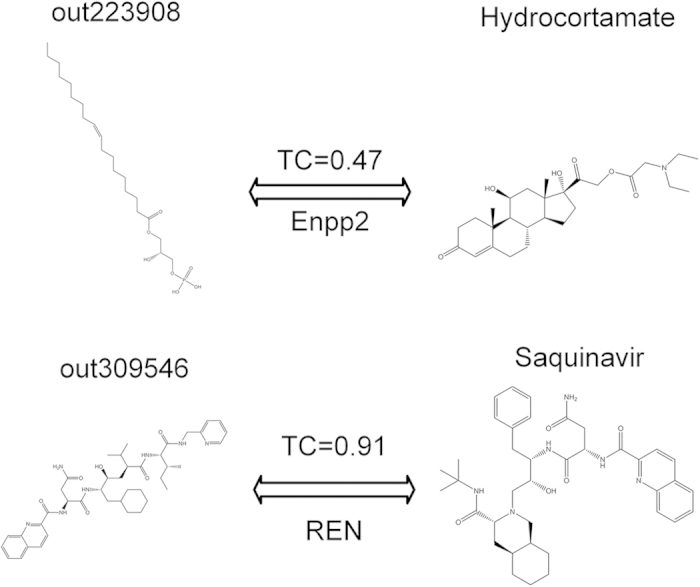
Non-intuitive (Hydrocortamate) and straightforward (Saquinavir) WES prediction, with Tc values to closest references.

In order to further assess the ligand scaffold hopping (LSH) ability for WES model, we have compared the predicted ligands with those known ligands for the same targets. The results show a diversified structural scaffolds as shown in Table S2-3. This indicates that WES catches the relatively complete drug-binding features for a protein from the ensemble level not from its single ligand like 1NN method. For example, drug Hydrocortamate, which is predicted to modulate Enpp2 (Fig. 3), is only marginally similar to the known ligand sets (Tc value 0.47; Fig. 3). Clearly, those similar compounds are more easily identified by WES. For example, Saquinavir, closely resemble (Tc value 0.91; Fig. 3) to the ligand set of REN, is predicted to regulate REN (Fig. 3). The LSH analysis confirms the specificity of prediction for WES, which is important for drug repositioning for those known drugs in pharmaceutical researches.

### Experimental validation

To validate the practicability of WES model, we randomly selected Enpp2, Faah, PTGS2, PPARG, and REN, the five inflammation-related targets, and predicted their direct ligand-target interactions. The 24 top-scoring (hybrid-WES) and commercially available drug-target interactions (Table 3) were tested by the ligand-binding assays.

Here, the ligand-target affinities are calculated by IC_50_ values, and the ligands were then classified as strong (IC_50_ < 1 μM), moderate (1 μM ≤ IC_50_ < 10 μM), weak (10 μM ≤ IC_50_ < 100 μM), or non-binders (IC_50_ ≥ 100 μM) according to Regina S. Salvat *et al.*^20^. In this work, the IC_50_ ≤ 10 μM is defined for binders for building the training dataset. Clearly, this criteria is strict, as we believe that a more strict strategy will be helpful to reduce data noises, since which were collected from various resources. Here, both the weak and strong binders were counted, resulting in a prediction ACC of 71% (17/24) for the experimental interactions predicted by the hybrid WES.

Perhaps the most compelling results are the test of the drugs against those targets to which they were not previously known to bind, so called drug repositioning (Table 3). By direct binding assay, we find Desmopressin is a new 1 μM antagonist of REN receptor, which was not reported previously. This is also consistent with the phenomenon for Treprostinil which is newly found to antagonize PPARG in a micromolar concentration range. Intriguingly, Esmolol is also observed to modulate PPARG, though it has been reported to act on ADRB1^21^.

## Discussion

The decoding of drug direct targets is of great importance in drug repositioning and discovery, but it is laborious and costly. Hence, a reliable computational approach for drug direct target prediction would be of significant values. In this study, we propose a new WES algorithm which exhibits reasonable reliability in discriminating direct interactions and non-interactions with a well specificity and sensitivity (AUC = 0.85), internal, external and experimental test accuracies of 78%, 70% and 71%, respectively.

Attention needs to be particularly paid to two steps in construction of the WES algorithm. First, the bulk of features have little to do with the pharmacological properties of a ligand. In order to identify the pharmacology-related features, we weighted the structural features based on statistical tests and optimization analysis in a framework of ensemble. This step not only reduces dimensionality of the descriptor set, but also eliminate data noise.

Second, most ligands are dissimilar with each other even they target to the same protein. Thus traditional single molecule similarity-based methods may be insufficient to predict the complex drug-target interactions. Here, we introduced the ensemble concept to assure the model to predict a compound activity not because of its similarity with certain compound in the training set, but of its similarity with the whole feature of an ensemble. Compared with the 1NN model, which judges the probability of a drug targeting to a protein based only on the maximum similarity to a reference ligand, the WES algorithm has more generalization ability in predicting those scaffold-hopping ligands.

## Methods

### Data sets

We obtained 822,643 protein-ligand pairs (PLPs) with information of inhibitory (Ki), IC_50_ values and protein sequences from the BindingDB database, including 5,311 proteins and 490,282 ligands, respectively. K_i_ is the concentration of an inhibitor that is required to decrease the maximal rate of the reaction by half. IC_50_ is a measure of the effectiveness of a substance in inhibiting a specific biological or biochemical function. To obtain a reliable data set, we filtered the PLPs with the following steps: (1) deleting the redundant PLPs based on the protein sequences and the ligand Inchkey; (2) removing the PLPs of which K_i_ and IC_50_ values are unavailable or the average value of them larger than 10 μM; (3) expunging the smaller ligand-set sized protein that overlaps more than 60% ligands with another protein; (4) excluding those ligands whose Tanimoto similarity is larger than 0.75 in the ligand set of one protein; (5) deleting the proteins whose ligand number is less than 5. As a result, 1788 proteins and 68,777 ligands that constituted 98,327 PLPs were obtained as the positive set. The negative set was constructed by a random generation of the same number of relations that do not overlap with those positive interactions. The two datasets are then used for training the models. All the data can be download from our website related with this work (http://lsp.nwsuaf.edu.cn/tcmsp.php).

## Construction of feature matrix.

### 

CDK Fingerprint matrix. Ligands were represented by 1,024-bit chemical hashed fingerprints, which were computed using the CDK with default 2D parameters. The CDK is a scientific, LGPL-ed library for bio-informatics and chemi-informatics and computational chemistry written in Java. Taking the ligand set of a protein *j* constituted by *n*_j_ ligands, an initial matrix *P* = {*F*^(j)^} (*n*_j_ × 1024) was generated to represent the protein, where 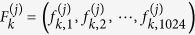 is the binary fingerprint vector of ligand *k*. To investigate which feature fit of the fingerprint has a higher contribution rate in distinguishing one protein from the others, we weighted each feature based on the significance (by *P*-value using one-sided Fisher’s exact test) of overrepresentation against the background incidence of the feature in respective protein. The *P*-values are adjusted to control for multiple hypothesis tests, yielding *q*-values. The weight for each feature was then computed using the following formula:


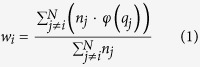


where 
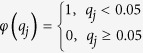
, *N* is the number of total proteins in the training set. We used *q* = 0.05, the generally considered statistically significant threshold, as it ensures a reasonable discrimination of the feature weights (Figure S1).

### Dragon Fingerprint matrix

In addition, ligands were also represented by 1,664 Dragon descriptors (http://www.talete.mi.it/index.htm). As a professional software package, Dragon calculates molecular descriptors frequently used to evaluate the molecular structure-activity relationship. Taking the ligand set of a protein *j* constituted by *n*_*j*_ ligands, an initial matrix *P* = {*D*^*(j)*^} (*n*_*j*_ × 1664) is generated to represent the protein, where 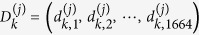. All *d*_*k,i*_ were standardized according to the equation of 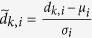 , where *μ*_*i*_ and *σ*_*i*_ are the mean and standard deviation of ligand *k*, respectively. To recognize those features that can signally differentiate these proteins, we weighted each feature based on non-parametric Wilcoxon Sum Rank Test. The *P*-values are adjusted to control multiple hypothesis testing, yielding q-values. The weight for each feature was then computed using equation (1).

### Model building

Firstly, for a protein *j*, we selected m_j1_ and m_j2_ highest weighted features from the CDK and Dragon descriptors, respectively; then the protein *j* was represented by the feature matrices *P* = {*F*^(*j*)^} (*n*_*j*_ × *m*_*j1*_) and *P* = {*D*^*(j)*^} (*n*_*j*_ × *m*_*j2*_); finally, the fingerprint-Dragon based weighted similarity scores between two ligand (*l*_1_, *l*_2_) were expressed as


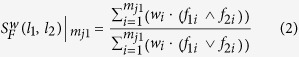


where ∧ indicts the Boolean operator “AND”, whereas ∨ represents the Boolean operator “OR”, respectively.


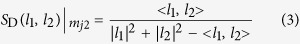


In equation (3), <·,·> denotes the inner product, whereas |·| represents the module, respectively.

The feature (CDK and Dragon) number m of a protein ligand set was determined by the optimization model (equation 4).


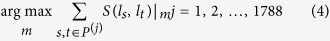


In order to obtain a good estimate of the overall similarity with the ligand set (ensemble), we first defined a *raw score* for this ligand by summing its weighted similarity relative to the ligand set of protein *j* with *S*_*i*_ ≥ *S*_*cut*_.


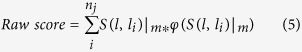


where 
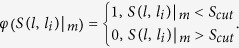


The threshold *S*_*cut*_ was determined by retrospective cross-fold analysis. Unlike WES, SEA chooses *S*_*cut*_ to meet that the random *Z score* is consistent and enriches for a BLAST-like background probability distribution. Actually, by sampling across the range of *S*_*cut*_ choices, we chose the threshold that will lead to the highest ROC AUC, resulting in a similarity threshold. The scores below the threshold were discarded which do not contribute to the overall similarity.

Then, a model of the distribution of random *raw scores* was developed and fitted. Random *raw scores* were calculated by comparing a randomly selected ligand set (size = 50) to the ligand set of each protein. Therefore, we can acquire the mean (*μ*) and standard deviation (*σ*) of the 50 random *raw scores*. And the normalized *raw score*, annotated as *Z score*, can be represented as equation (6):


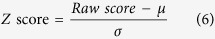


The calculation process of *Z score* is as follows:

1. For a protein *j*, choose 50 ligands at random from all ligands and calculate the mean and standard deviation values of *raw scores* at different similarity thresholds (*S*_*cut*_) with step size 0.01, where 0 < *S*_*cut*_ < 1. Store all calculated mean values (*μ*_*j*_ = {*μ*_*j*1_,…, *μ*_*j*100_}) and standard deviation values (*σ*_*j*_ = {*σ*_*j*1_,…, *σ*_*j*100_}), along with the set size of the protein *j*.
2. For each *S*_*cut*_, plot the set size of protein ligand vs all *μj*(*S*_*cut*_) and *σj*(*S*_*cut*_) scores, respectively; and then the linear regression was applied to determine the equations of *μ*_*j*_ and *σ*_*j*_. Typically, equations *y*_*μ*_ = *α*_1_*x* + *β*_1_ and *y*_*σ*_ = *α*_2_*x* + *β*_2_ are appropriate for standardizing the Raw sores. Given the normalized equation (6), calculate the *Z score*. If a new drug–target interaction has a *Z score* above a threshold, it will be treated as a direct interaction. The threshold above which the highest F1 score was achieved in LOOCV was used to make predictions (equation 7).

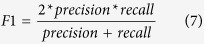


where precision is the ratio of the number of true positives to the number of predicted positives and recall is the ratio of the true positives which are correctly identified.

### Z score integration

To depict the likelihood of a ligand binds to a specific protein, we integrated the *Z scores* into a likelihood value by the Bayesian network method, so called the hybrid model in this work. The likelihood was defined as:


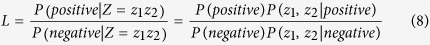


where *P*(Z = *z*_1_,*z*_2_|*C* = *c*) indicates the probability of *Z score* scored *z*_*1*_ or *z*_*2*_ in class *c*, and *z*_*1*_ and *z*_*2*_ represent the CDK and Dragon *Z scores*, respectively.

In addition, we evaluated the conditional probability by the multivariate kernel density estimation approach, which is a nonparametric technique for density estimation through the following formula:


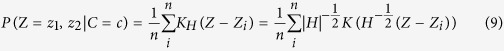


where, 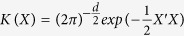 is the Gaussian kernel, *d* is the dimensionality of vector X, (*d* = 2); n is the number of data samples in class *c*, H is the bandwidth (or smoothing) *d* × *d* matrix which is symmetric and positive definite. And a ligand is considered to incorporate into a protein when the L value is greater than threshold θ, which is the same as the threshold of *Z score*.

### Performance evaluation

The WES model was evaluated and verified with LOOCV. In details, the WES algorithm is applied once for each interaction, using all other interactions as a training set and using the selected interaction as a single-item test set. Several parameters, ACC (equation 10), SEN (equation 11), SPE (equation 12) and PRE (equation 13), were used to measure the accuracy of overall, positive prediction, negative prediction and the positive predictive value of the model, respectively.


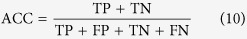



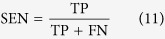



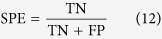



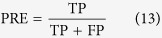


here, the TP, TN, FP and FN represent the number of true-positives, true-negatives, false-positives and false-negatives, respectively.

### Comparison to a 1NN model

We evaluated two 1NN models, using either CDK or Dragon fingerprints. For a drug, it was compared to all known ligands of a target. The highest Tc value between the querying drug and known ligands was assigned to the drug-target pair. For each drug, we identified the lowest Tc value that yielded valid WES predictions using the respective fingerprint and collected all drug-target pairs with Tc scores above that threshold. We calculated an adjusted hit rate (equation 14):


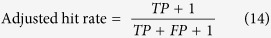


The additional count for both numerator and denominator distinguishes cases where no predictions were confirmed.

#### External data validation for binding and non-binding data

To examine the generalization ability of WES, we manually collected the direct binding data in PDB and non-binding data in BindingDB (see details in Results).

### Experimental validation

Molelues like Bleomycin, Pasireotide, Fingolimod, Hydrocortamate, Vancomycin, Alpha-Linolenic Acid, Pentagastrin, Roxatidine acetate, Alpha-Linolenic Acid, Mupirocin, Rimonabant, Pravastatin, Treprostinil, Esmolol, Cetrorelix, Carfilzomib, Saquinavir, Lopinavir, Indinavir, Ritonavir, Desmopressin, and Felypressin were purchased from Yitai Technology Ltd. (Wuhan, China). Enpp2 (Autotaxin Inhibitor Screening Assay Kit), Faah (FAAH Inhibitor Screening Assay Kit), PTGS2 (COX Inhibitor Screening Assay Kit), PPARG (PPARγ Ligand Screening Assay Kit), and REN (Renin Inhibitor Screening Assay Kit) were purchased from Cayman Chemical, Ann Arbor, MI, USA. All drugs were dissolved in DMSO and freshly prepared due to the loss of activity under long-term storage. The activity of targets was detected according to manufacturer’s instructions. IC_50_ values were determined using the Bliss method according to the eight data points per drug. The same drug-target interaction was repeated independently three times to obtain a mean IC_50_ value and its standard deviation.

## Additional Information

**How to cite this article**: Zheng, C. *et al.* Large-scale Direct Targeting for Drug Repositioning and Discovery. *Sci. Rep.* **5**, 11970; doi: 10.1038/srep11970 (2015).

## Supplementary Material

Supplementary Information

## Figures and Tables

**Table 1:** Performance of the WES method.

Data set	Data type	ACC	SPE	SEN	PRE	AUC
Feature classes	Dragon + CDK	0.78	0.71	0.85	0.74	0.85
	Dragon	0.75	0.63	0.85	0.70	0.83
	CDK	0.74	0.66	0.82	0.71	0.80
Target classes	Ion channel	0.75	0.69	0.80	0.72	0.84
	Membrane receptors	0.79	0.73	0.85	0.76	0.86
	Transcription factor	0.80	0.74	0.85	0.77	0.86
	Transporter	0.79	0.69	0.89	0.74	0.87
	Enzyme	0.78	0.71	0.86	0.75	0.86
External validation	PDB (IC50 < 10) and BindingDB (IC50 > 500 μM)	0.70	0.70	0.71	0.32	0.75

**Table 2:** Statistics of the prediction performance.

Data	Method	AUC	Sensitivity	Specificity
Enzyme	Nearest profile	0.77	0.54	1
	Weighted profile	0.81	0.39	0.99
	Bipartite Graph learning	0.9	0.57	1
	WES	0.86	0.54	1
Ion channel	Nearest profile	0.75	0.17	1
	Weighted profile	0.81	0.24	1
	Bipartite Graph learning	0.85	0.27	1
	WES	0.84	0.26	1
GPCR/Membrane receptors	Nearest profile	0.73	0.16	0.99
	Weighted profile	0.74	0.15	0.99
	Bipartite Graph learning	0.9	0.23	1
	WES	0.86	0.22	1
Transcription factor	Nearest profile	–	–	–
	Weighted profile	–	–	–
	Bipartite Graph learning	–	–	–
	WES	0.86	0.27	0.99
Transporter	Nearest profile	–	–	–
	Weighted profile	–	–	–
	Bipartite Graph learning	–	–	–
	WES	0.87	0.26	0.99

**Table 3:** IC_50_values for the 24 top-scored direct interactions.

NO.	Target gene name	Drug name	IC_50_ (μM):mean ± SD
1	Enpp2	Bleomycin	71.39 ± 3.2
2	Enpp2	Pasireotide	73.28 ± 9.7
3	Enpp2	Fingolimod	114.78 ± 5.8
4	Enpp2	Hydrocortamate	218 ± 6.4
5	Enpp2	Vancomycin	68.54 ± 7.0
6	Faah	Alpha-linolenic acid	53.86 ± 11.5
7	Faah	Pentagastrin	222.61 ± 8.3
8	Faah	Roxatidine acetate	34.53 ± 1.5
9	Faah	Alpha-linolenic acid	43.86 ± 15
10	PTGS2	Mupirocin	123.39 ± 7.4
11	PTGS2	Rimonabant	138.37 ± 3.5
12	PTGS2	Pravastatin	199.13 ± 12.3
13	PPARG	Treprostinil	69.01 ± 17.5
14	PPARG	Esmolol	40.77 ± 6.5
15	PPARG	Propafenone	36.44 ± 13.2
16	REN	Pentagastrin	3.45 ± 4.8
17	REN	Cetrorelix	156.44 ± 3.7
18	REN	Carfilzomib	22.01 ± 6.4
19	REN	Saquinavir	69.1 ± 4.2
20	REN	Lopinavir	49.35 ± 10.3
21	REN	Indinavir	44.32 ± 12.1
22	REN	Ritonavir	26.11 ± 13.2
23	REN	Desmopressin	1 ± 2.6
24	REN	Felypressin	4.5 ± 7.1
