# Supplementary material for: Large-scale Direct Targeting for Drug Repositioning and Discovery
Source: Sci Rep. 2015 Jul 9;5:11970. doi: 10.1038/srep11970 (PMC4496667; doi:10.1038/srep11970)
Supplement: Supplementary Information [file srep11970-s1.pdf]

Large-scale Direct Targeting for Drug Repositioning and Discovery

Chunli Zheng[1,+], Zihu Guo[1,+], Chao Huang[1,+], Ziyin Wu[1], Yan Li[2], Xuetong Chen[1], Yingxue Fu[1], Jinlong Ru[1], Piar Ali Shar[1], Yuan Wang[3], Yonghua Wang[1*]

[1]Bioinformatics Center, College of Life Sciences, Northwest A&F University, Yangling, Shaanxi, 712100, China.

[2]Department of Materials Science and Chemical Engineering, Dalian University of Technology, Dalian, Liaoning, 116000, China.

[3]Department of Pathology and MCW Cancer Center, Medical College of Wisconsin, Milwaukee, WI 53226. USA.

*corresponding author yh_wang@nwsuaf.edu.cn

[+]these authors contributed equally to this work

Supplementary Figures

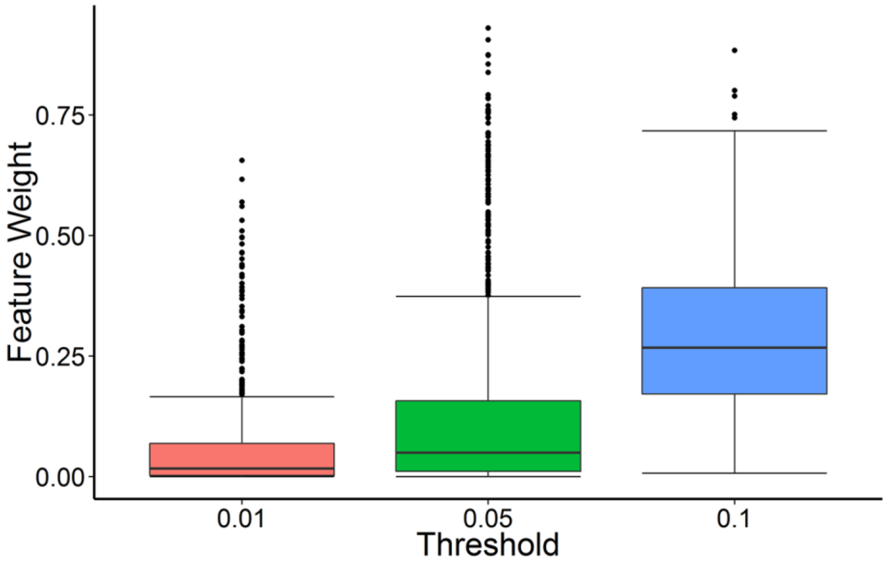

Figure S1. Feature weights of different proteins at thresholds 0.01, 0.05 and 0.1.

Supplementary Tables

Table S1. Feature analysis of WES

Model	ACC	SPE	SEN	PRE	F1
**unweighted Dragon model**	0.71	0.69	0.73	0.7	0.72
**unweighted CDK model**	0.69	0.66	0.72	0.68	0.70
**unweighted Hybrid model**	0.73	0.71	0.75	0.72	0.73
**weighted Dragon model**	0.75	0.63	0.85	0.70	0.77
**weighted CDK model**	0.74	0.66	0.82	0.71	0.76
**weighted Hybrid model**	0.78	0.71	0.85	0.74	0.79

Table S2. The scaffold hopping information for direct interactions.

Target	Known ligand		Predicted ligand		Tc
	ID	Structure	Name	Structure	
Enpp2	out327825	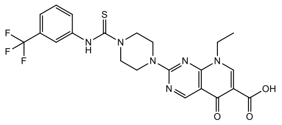	Bleomycin	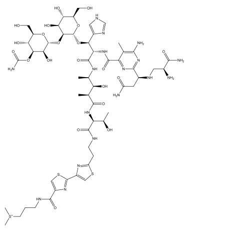	0.52
Enpp2	out360500	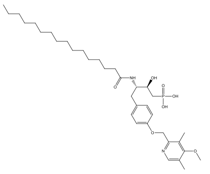	Pasireotide	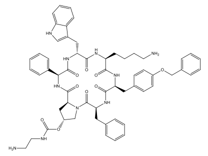	0.74
Enpp2	out327837	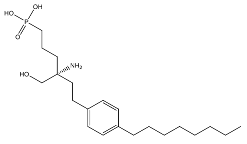	Fingolimod	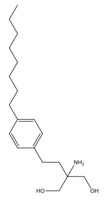	0.91
Enpp2	out223908	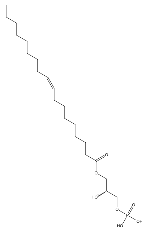	Hydrocortamate	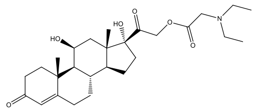	0.47
Enpp2	out350307	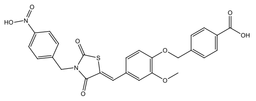	Vancomycin	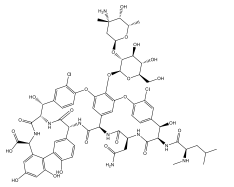	0.55
Faah	out207912	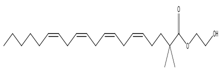	Alpha-linolenic acid	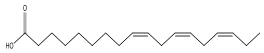	0.67
Faah	out274504	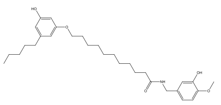	Aliskiren	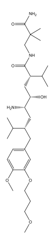	0.80

Table S2. Continued

Target	Known ligand		Predicted ligand		Tc
	ID	Structure	Name	Structure	
Faah	out22592	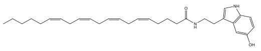	Pentagastrin	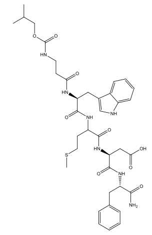	0.70
Faah	out346470	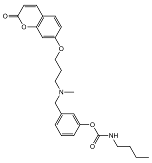	Roxatidine acetate	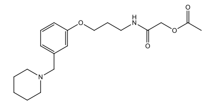	0.80
PTGS2	out301501	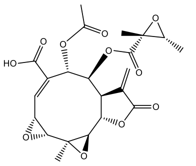	Mupirocin	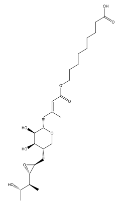	0.78
PTGS2	out149313	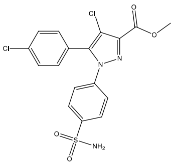	Rimonabant	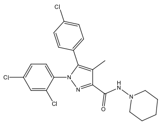	0.71
PTGS2	out296157	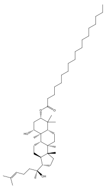	Pravastatin	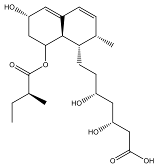	0.70
PPARG	out243439	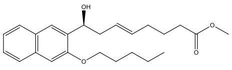	Treprostinil	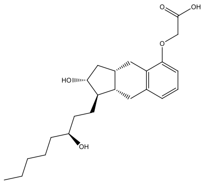	0.81
PPARG	out446136	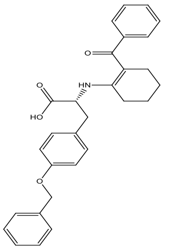	Esmolol	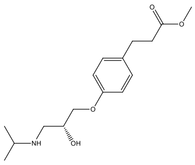	0.79

Table S2. Continued

Target	Known ligand		Predicted ligand		Tc
	ID	Structure	Name	Structure	
PPARG	out446136	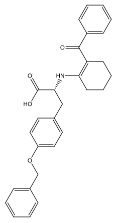	Propafenone	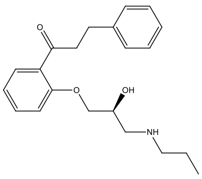	0.79
REN	out122088	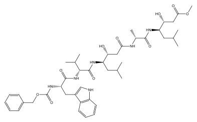	Pentagastrin	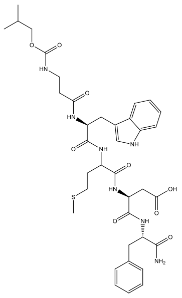	0.85
REN	out110558	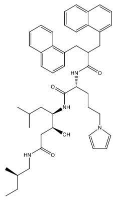	Cetrorelix	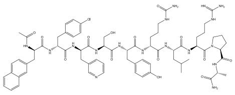	0.79
REN	out107264	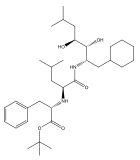	Carfilzomib	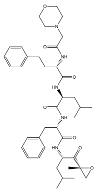	0.82
REN	out309546	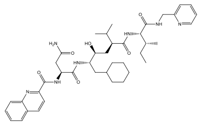	Saquinavir	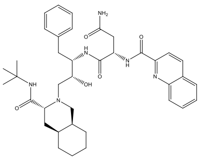	0.91
REN	out313603	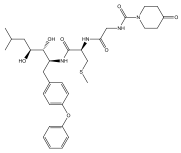	Lopinavir	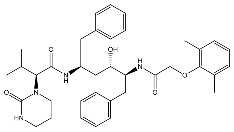	0.84
REN	out132195	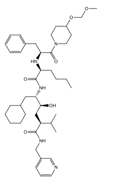	Indinavir	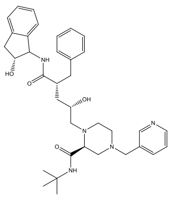	0.87

Table S2. Continued

Target	Known ligand		Predicted ligand		Tc
	ID	Structure	Name	Structure	
REN	out140166	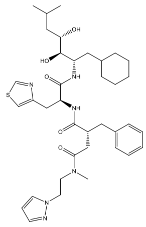	Ritonavir	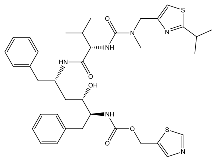	0.82
REN	out109037	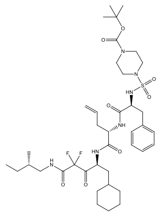	Felypressin	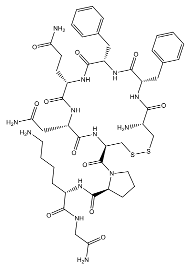	0.76
REN	out124454	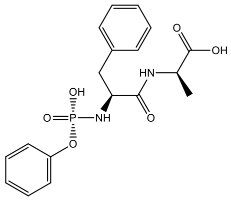	Desmopressin	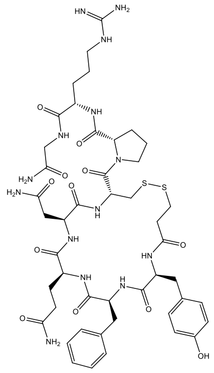	0.72

Table S3. The InChIKey information for the known ligands of targets in Table S2.

ID	InChIKey
out327825	USYVBPXJSBDUTJ-UHFFFAOYSA-N
out327837	PQADYNLAFBSVRI-OAQYLSRUSA-N
out223908	WRGQSWVCFNIUNZ-GDCKJWNLSA-N
out350307	ATSKXWVQALVDSP-QRVIBDJDSA-N
out207912	WJKSCUWBYPTSMN-GKFVBPDJSA-N
out274504	BBTJHAUUGXYTQE-UHFFFAOYSA-N
out22592	QJDNHGXNNRLIGA-DOFZRALJSA-N
out346470	MTHAAFJWKDKWDN-UHFFFAOYSA-N
out301501	YOARICMECKZMHY-XIMYPQPTSA-N
out149313	MEBSTASXWKTLJD-UHFFFAOYSA-N
out296157	BCRQTNOYBAWCDF-XRYSTRTLSA-N
out243439	QZQQRILMZZQVCH-HVPBSWBLSA-N
out446136	JIQZXPNJCFZXNY-MHZLTWQESA-N
out446136	JIQZXPNJCFZXNY-MHZLTWQESA-N
out122088	OBRHZWCYAVPXND-ZAIMBGABSA-N
out110558	CPSOBBIPRDHWCX-MMZKXQSJSA-N
out107264	CKOVQLLOHXSFKN-VFFRCKCKSA-N
out309546	ACLGFPBLVWSDHZ-TZLBSOBUSA-N
out313603	IDXYOZNTCOWYTI-VNDOHOEKSA-N
out132195	ZCTACFQVPLBIDT-HECCNADXSA-N
out140166	XYAOQVSQLNESBZ-AJISXOSTSA-N
out124454	WTHGJISEXACGFF-CJNGLKHVSA-N
out109037	MNARPUVTMSONDZ-QJANCWQKSA-N
